# Climate Denial Fuels Climate Change Discussions More Than Local Climate-Related Disasters

**DOI:** 10.3389/fpsyg.2021.682057

**Published:** 2021-08-26

**Authors:** Miti Shah, Sarah Seraj, James W. Pennebaker

**Affiliations:** Department of Psychology, The University of Texas at Austin, Austin, TX, United States

**Keywords:** climate change, climate denial, collective action, political leaders, natural disasters

## Abstract

Most scientists agree that climate change is the largest existential threat of our time. Despite the magnitude of the threat, surprisingly few climate-related discussions take place on social media. What factors drive online discussions about climate change? In this study, we examined the occurrence of Reddit discussions around three types of climate-related events: natural disasters (e.g., hurricanes, wildfires), political events (i.e., 2016 United States Presidential election), and policy events (i.e., United States’ withdrawal from Paris Climate Agreement, release of IPCC report). The objective was to understand how different types of events influence collective action as measured by discussions of climate change. Six large US cities were selected based on the occurrence of at least one locally-relevant natural disaster since 2014. Posts (*N* = 4.4 million) from subreddits of the selected cities were collected to obtain a six-month period before and after local natural disasters as well as climate-related political and policy events (which applied equally to all cities). Climate change discussions increased significantly for all three types of events, with the highest discussion during the 2016 elections. Further, discussions returned to baseline levels within 2 months following natural disasters and policy events but continued at elevated rates for up to 4 months following the 2016 elections. The findings suggest that collective discussions on climate change are driven more by political leaders’ controversial positions than life-threatening local natural disasters themselves. Implications for collective action are discussed.

## Introduction

People may lack a sense of urgency about climate change for several reasons – the topic is abstract, changes in climate are slow-moving and not disruptive to people’s daily lives (Gifford, 2011). There are many objective signs of climate change, such as increased frequency and intensity of heatwaves, droughts and wildfires (U.S. Global Change Research Program, Wuebbles et al., 2017). There is some evidence that extreme weather events increase people’s engagement in climate change issues (Bergquist et al., 2019). Google searches for climate change increased in the months following tropical cyclones in affected regions (Lang and Ryder, 2016), and Twitter mentions increased after Hurricanes Irene and Sandy and Snowstorm Jonas (Roxburgh et al., 2019). It is possible that the occurrence of these events makes climate change more concrete in the minds of people instead of an abstract phenomenon far into the future, leading to more engagement.

While studies have looked at climate change engagement following natural disasters, it has not been compared with engagement following other relevant events. Climate change has become a deeply political issue in the United States. During his presidency, Donald Trump was well known for his skepticism on climate change (Matthews, 2017). Zawadzki et al. (2020) found that people expressed higher intentions to engage in climate-friendly behaviors after the 2016 elections. With a climate denying leader, people may be motivated to engage in collective action for climate change at a greater intensity. Certain policy changes and treaties can also affect people’s concerns about climate change. For example, in 2017, the US’ withdrawal from the Paris Climate Agreement, an international climate treaty, caused shockwaves in the news cycle. Similarly, exposure to IPCC (Intergovernmental Panel on Climate Change) reports has been linked to greater perceived threat from climate change and increased climate concern (Ogunbode et al., 2020).

One way to understand public engagement of climate-related topics is by tracking the ways people talk about climate change on social media. Reddit contains over 180,000 online discussion communities (subreddits) on a variety of topics (WebsiteBuilder, 2020) providing an opportunity to track natural conversations over time. To explore the circumstances under which people are most likely to engage in climate change conversations, we analyzed climate discussions on Reddit city subreddits surrounding three types of events: climate-linked extreme weather, the 2016 US elections and international climate policy events. It was predicted that people would be most likely to discuss climate change around natural disaster events, compared to the other two types of events.

## Method

A total of 4.4 million comments were collected from six different city subreddits. Cities were selected based on the occurrence of at least one major natural disaster from 2014 to 2019. For example, r/houston was chosen because of Hurricane Harvey in August 2017, r/LosAngeles because of the Thomas wildfire in December 2017 and the Camp Fire wildfire in November 2018 (see details in Table 1). For every city, discussions around two other types of events were also collected: the 2016 United States elections and climate-related policy events (2017 United States withdrawal from Paris Climate Agreement and 2018 release of special IPCC report).

**TABLE 1:** Details of city subreddits and related natural disasters.

**City**	**Subreddit**	**Subscribers (as of Nov 2020)**	**Percentage of subscribers by city population^1^**	**Total comments in selected time period**	**Disaster event**
Houston	r/houston	179,000	7%	1,150,561	Hurricane Harvey, August 2017
Miami	r/Miami	56,600	12%	149,310	Hurricane Irma, September 2017
Los Angeles	r/LosAngeles	226,000	6%	1,213,475	Camp Fire, November 2018 Thomas Fire, December 2017
San Diego	r/sandiego	130,000	9%	473,709	Carr Fire, August 2018
Boston	r/boston	192,000	27%	1,120,644	Blizzard, February 2015
Dallas	r/Dallas	135,000	10%	390,973	Heatwave, July 2018

*^1^City population estimates were obtained from the U.S. Census Bureau (2019). Percentage of subscribers by city population was calculated by dividing number of subscribers per city subreddit by the estimated total population.*

To identify when discussions on climate change emerged in the subreddits, a climate change dictionary was developed to computationally capture references to climate change. It was based on an extensive search by two independent coders of related terms used by Reddit users (e.g., warming oceans, CO2 emissions) as well as commonly used terms in the media (e.g., climate crisis, climate action). The dictionary was entered into the topic modeling tool Meaning Extraction Helper (Boyd, 2016) to identify posts that mentioned one or more of the terms. A binary count of 1 or 0 was given to each post depending on whether it used a climate change term or not. For example, if a post made by a user contained the terms “climate change” and “global warming,” it would be given a binary score of 1 and if a post did not contain any climate change terms then it would be given a score of 0. In this way, we calculated the raw counts of climate mentions per month, i.e., the total number of posts by users that contained climate change terms.

The percentage of posts containing climate terms was calculated by dividing the raw count of comments on climate change by the total number of comments on each subreddit on any given day. The results were aggregated by month to get a total percentage of comments on climate change per month for each subreddit.

In order to understand the rate of discussions around natural disasters, a period of 13 months around the disaster was selected: 6 months before and after the disaster, as well as the month of the disaster. Monthly percentages of comments were aggregated across all natural disasters to compute average percentages of discussions per month surrounding natural disasters. In the final step, we aggregated the data into bi-monthly averages of discussions except for the actual month of the natural disaster. This was done to reduce noise and observe discernible patterns in climate change discussions. The same process was repeated with the other two types of events, the 2016 elections and climate-related policy events. Two-proportion *Z*-tests were used to statistically compare discussion rates between time points and between each type of event.

## Results

**FIGURE 1:**
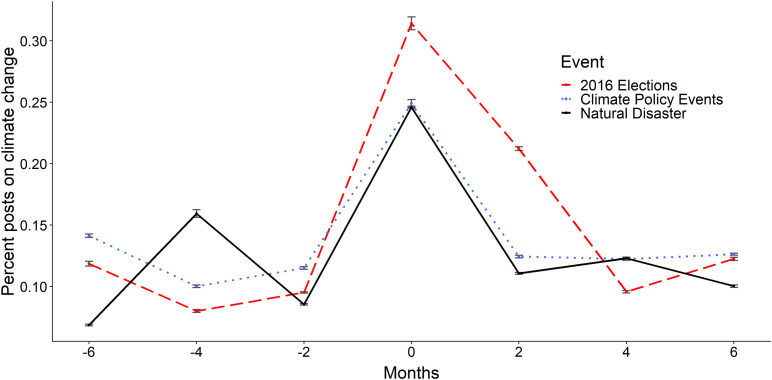
Bi-monthly rate of discussion on climate change surrounding (1) city-relevant natural disasters (e.g., Hurricane Harvey; black solid line), (2) the 2016 United States Presidential elections (red dashed line), and (3) climate-related policy events (e.g., IPCC special report release; blue dotted line). Note: Month 0 refers to the month of the event occurring.

Bi-monthly percentages of posts on climate change were computed for each type of climate event. As depicted in Figure 1, discussions increased during all three types of events with the highest rate during the 2016 presidential elections. In November 2016, discussions increased significantly from the previous 2 months [χ^2^(1) = 6.45, *p* = 0.011, and *h* = 0.03] with 0.31% of total conversations being on climate change. Similarly, during natural disasters and policy events, climate discussions accounted for 0.25% of total discussions, with a significant increase from baseline for natural disasters [χ^2^(1) = 6.69, *p* = 0.010, and *h* = 0.02] but not for policy events [χ^2^(1) = 2.58, *p* = 0.108, and *h* = 0.02]. We did not find a significant difference in discussion rates between the three types of events in Month 0.2 months after each event, discussion rates were significantly higher following the 2016 elections compared to natural disasters [χ^2^(1) = 4.24, *p* = 0.040, and *h* = 0.02] and climate policy events [χ^2^(1) = 4.10, *p* = 0.043, and *h* = 0.02]. There was no difference in the discussion rates between natural disasters and climate policy events 2 months later. Discussions following these two events tapered off within the next 2 months, reaching close to baseline levels but continued at higher rates for up to 4 months after the 2016 elections.

### Robustness Checks

We conducted two additional sets of analyses to check the robustness of the findings. To check if the increased discussion rates were in support of climate action and not due to increased skepticism, a random subset of posts per event (*n* = 120) was coded. Overall, the majority of posts (62.2%) on climate change for each event were in support of climate action (see Supplementary Information I). We also checked to see if increased discussion rates corresponded proportionally to the number of authors discussing climate change. In the month of the event occurring, approximately the same percentage of users discussed climate change, but a much higher rate of users continued to discuss climate change 2 months after the 2016 elections (see Supplementary Information II). This analysis indicated that although the relative increase in users discussing climate change is similar across the three events, the volume of discussion is comparatively higher during the elections. Furthermore, and in line with our main finding, the discussion is sustained with a higher percentage of users discussing climate change 2 months after the elections whereas for natural disasters and climate policy events, percentage of users discussing climate change reaches baseline levels within 2 months.

## Discussion

Across the six cities that experienced at least one major natural disaster, people were more likely to discuss climate change following the 2016 elections than in the aftermath of their own community’s climate-related disaster. On the surface, these findings appear paradoxical. A natural disaster that hits your community, after all, is salient, personal, and could recur in the future. Even if people are convinced that climate change contributed to the event, why don’t they engage in climate-related discussions and collective actions at higher rates? And why do the same communities become more mobilized about climate change with the election of a climate-denying leader?

One key in understanding this paradox is to appreciate how people perceive cause and effect. Humans have evolved exquisite ways to detect and understand which actions result in effective changes in their environment. Putting on a hat, getting an umbrella, or even building a shed with a roof can protect us from rain. As the projected outcomes of our actions shift from the here and now to the distant future, the less we can appreciate them. Several compelling projects on affective forecasting suggest that our imagined futures are based on our current emotional states (Gilbert, 2011; Quoidbach et al., 2013). We immediately grasp clear and present dangers and are highly sensitive to threats from other human beings. However, impersonal threats that may not be realized for decades are beyond most people’s immediate concern. People whose houses have been damaged by a hurricane tend to focus on rebuilding and preventing similar damage in the future. They are not denying the reality of climate change. Rather, it simply is not relevant to their immediate problems.

From a psychological perspective, it makes sense why people become more involved in climate change discussions surrounding elections than natural disasters. Political events put a human face on the issue and make the climate issue a more personal, immediate, and actionable topic. That the global temperature will increase by 2 degrees Celsius over the next 100 years is conceptually difficult to grasp. Very few actions that individuals take in the wake of a natural disaster will affect this change. By focusing on salient leaders who violate people’s moral sensibilities about climate change, collective action is more likely to occur. In fact, psychological threats to people’s sense of freedom and control from others are well known to provoke strong reactions (Brehm, 1966). For example, reading a message about scientific consensus on climate change produces more reactance in climate skeptics, especially Republicans, and little, or no reactance in climate believers (Ma et al., 2019).

The implications for understanding collective action in affecting discussions of large-scale and largely theoretical topics such as climate change are clear, and unsettling. People must perceive threats as personal, imminent, actionable, and likely aimed directly at them by others. Despots and savvy advertising agencies have exploited these human tendencies over the course of history. The ethical challenge for well-meaning and noble leaders is to use these psychological methods in transparent ways.

There are some limitations to this study. Since the three types of events are different in their duration, intensity, and impact, they might generate a variety of reactions from the public beyond increased engagement on Reddit. So even if the event led to other collective action such as participating in a protest or donating money to relief funds, it does not necessarily get captured in our dataset. It is also possible that Trump is an anomaly in how he led to an increase in climate action. He was not only a divisive leader but also garnered a lot of media attention for his controversial opinions. A different leader might not have caused climate engagement in the same way. Future studies should look at other controversial leaders who may violate people’s moral sensibilities and how that affects people’s motivation to engage in collective action.

## Data Availability Statement

All the Reddit datasets used in this study are publicly available at the links below (Accessed: 2020-07-21). Submissions: https://files.pushshift.io/reddit/submissions/, Comments: https://files.pushshift.io/reddit/comments/. The processed datasets used in this study and the associated code can be found at the following link: https://osf.io/urf2n/.

## Author Contributions

MS extracted and analyzed the Reddit data. MS, SS, and JWP designed the study and wrote the manuscript. All authors contributed to the article and approved the submitted version.

## Conflict of Interest

The authors declare that the research was conducted in the absence of any commercial or financial relationships that could be construed as a potential conflict of interest.

## Publisher’s Note

All claims expressed in this article are solely those of the authors and do not necessarily represent those of their affiliated organizations, or those of the publisher, the editors and the reviewers. Any product that may be evaluated in this article, or claim that may be made by its manufacturer, is not guaranteed or endorsed by the publisher.
