# Supplementary material for: Climate Denial Fuels Climate Change Discussions More Than Local Climate-Related Disasters
Source: Front Psychol. 2021 Aug 26;12:682057. doi: 10.3389/fpsyg.2021.682057 (PMC8426507; doi:10.3389/fpsyg.2021.682057)
Supplement: Supplementary file 1 [file Data_Sheet_1.docx]

**Supplementary Information for**

Climate Denial Fuels Climate Change Discussions More Than Local Climate-Related Disasters

Miti Shah, Sarah Seraj, James W. Pennebaker

The University of Texas at Austin

*Miti Shah

mitishah@utexas.edu

This file includes:

Supplementary text

Figure S1

Table S1

SI References

**Supplementary Text**

**This Supplementary Text file includes:**

**I. Climate Discussion Analysis**

**II. User Analysis**

**I. Climate Discussion Analysis**

In the main manuscript, the analysis is focused on the rate of discussions related to climate change. However, it is also instructive to look at the themes of discussions to understand if the increase in climate discussions is due to a corresponding increase in concern or skepticism related to climate change.

We randomly selected 120 posts on climate change during each type of event analyzed in our study (natural disasters, 2016 elections and climate policy events), with roughly 20 posts from each city subreddit (Miami, Houston, Boston, Los Angeles, San Diego and Dallas) per event. Each post was coded for whether it was in support of climate action (including concern for climate change) or skeptical of climate change. Posts that were neutral or with unclear positions were put into a separate category. The percentage of posts per category and per event can be seen in Table S1.

We found that for each event, the majority of posts indicated support for climate action. On average, 62.2% of posts were in support of climate action (including posts that showed concern related to climate change) whereas only 13.1% were skeptical of climate change. A slightly higher percentage, 24.7% of posts were either neutral or their position was unclear towards climate change (see examples of posts below Table S1).

**Table S1.** Climate change discussion rates by theme and type of event

| Type of posts on climate change | Natural Disaster | 2016 Elections | Climate Policy Events | Average % of posts on climate change |
| --- | --- | --- | --- | --- |
| In support | 72.5% | 54.2% | 60% | 62.2% |
| Skeptical | 18.3% | 12.5% | 8.3% | 13.1% |
| Neutral/Unclear | 9.2% | 33.3% | 31.7% | 24.7% |

**Examples of discussion posts by theme and type of event**

**Natural disaster**

**In support:** “If you agree that climate change is happening, then you have to agree that these storms came from climate change. Weather is a product of climate. Our climate is changing. Therefor, our weather is different.”

**Skeptical:** “It was snowing in my backyard, so climate change is bullshit”

**Neutral/unclear:** “Went to a forum on capitalism and climate change at a Unitarian Church after I saw the event on my Facebook feed posted by the Democratic Socialists on Los Angeles. Anyone else here go to Democratic Socialist type events?”

**2016 Elections**

**In support:** “I didn't mean to direct it just at you, I just had to throw some information out there for people who don't believe in climate change. I think that picking economics over our environment for future generations isn't good though”

**Skeptical:** “About as valid as your "wew, it's warm today, IRREVERSIBLE GLOBAL WARMING IS PROVEN, IT'S THE END OF DAYS!"I will repeat: weather fluctuates, dumbass.”

**Neutral/Unclear:** “Plastic bags have nothing to do with climate change. It is still an environmental nuisance though.”

**Climate Policy Events**

**In support:** “There was just released a report stating that we have about 12 years to really stave off major mechanisms of climate change, but yeah, give landmark status to an oil company.”

**Skeptical:** “That's such a bad way of looking at things. What regulation has been implemented recently that has caused the recent downturn in total carbon emissions? Waste naturally decreases as tech grows. It's been doing that since the Industrial Revolution. Companies are already innovating a way to lower emissions. Regulations aren't needed.”

**Neutral/Unclear:** “Nothing but words. He couldn't even specifically say what he would or could do to make it happen, because he has no fucking clue. He should have just said he believes in climate change instead, because that is all he means.”

That these posts are mostly in support of climate action further supports our findings by showing that concern for climate change increases following each type of event and is not a result of increased skepticism related to climate change. Furthermore, in coding these subsets of posts, we did not find any posts that may have been made from fake or bot accounts that have been linked to spreading political propaganda on certain subreddits [(Broderick, 2018)](https://www.zotero.org/google-docs/?fJvZYC).

**II. User Analysis**

Do climate discussions increase because more users are engaged in the discussions or because a small number of posters are simply more active? Our findings show that discussions related to climate change increase during each type of event and are sustained at higher rates following the 2016 elections. Presumably, this is because a higher number of users engage in climate change discussions due to increased concern about the climate. In SI-I, we show that the majority of posts are in support of climate action, i.e., higher rates of discussion are mostly due to increased concern for the climate. In this section, we checked if the elevated rates were driven by an increased number of Reddit users participating in discussions or simply by increased engagement of a static number of users.

For this, we performed a user analysis similar to our analysis of climate discussions over time. For each event, we extracted the number of distinct users posting about climate change per month, for 13 months surrounding the event. For example, in August 2017 when Houston was hit by Hurricane Harvey, we extracted the number of distinct users on r/Houston who posted about climate change in that month and also the total number of users posting on the subreddit for that month. Using that, we calculated the percentage of users discussing climate change per month compared to the total users active on the subreddits (making posts on other topics including climate change). In the final step, we calculated bi-monthly averages (see Figure S1).

We found that in the month of the event, similar percentages of users (~0.8%) discussed climate change for all three events. This means that about 0.8% of the total number of users per subreddit discussed climate change during each type of event. In our main finding, we saw that climate change discussions are relatively higher during the 2016 presidential elections compared to natural disasters or climate policy events. This supplementary analysis suggests that even though users discussed climate change at slightly higher rates during the 2016 elections, the relative increase in number of people who got engaged in climate change discussions is similar across all three events. Further, the percentage of users talking about climate change two months after the elections was much higher than the other two events where users have gone back to baseline levels. This further reinforces the main finding where we see that discussion rates are higher and sustained for longer following the 2016 elections, corresponding to a higher percentage of users who are discussing climate change even two months later.

Finally, we also checked to see how individual users were discussing climate change, that is, if certain users' activity may have caused the discussions rates to increase. However, we did not find any users who may have disproportionately contributed to the climate change discussions. While some users were relatively more active and contributed more posts on climate change, the number of posts per user and their distribution was similar across all three events.

**
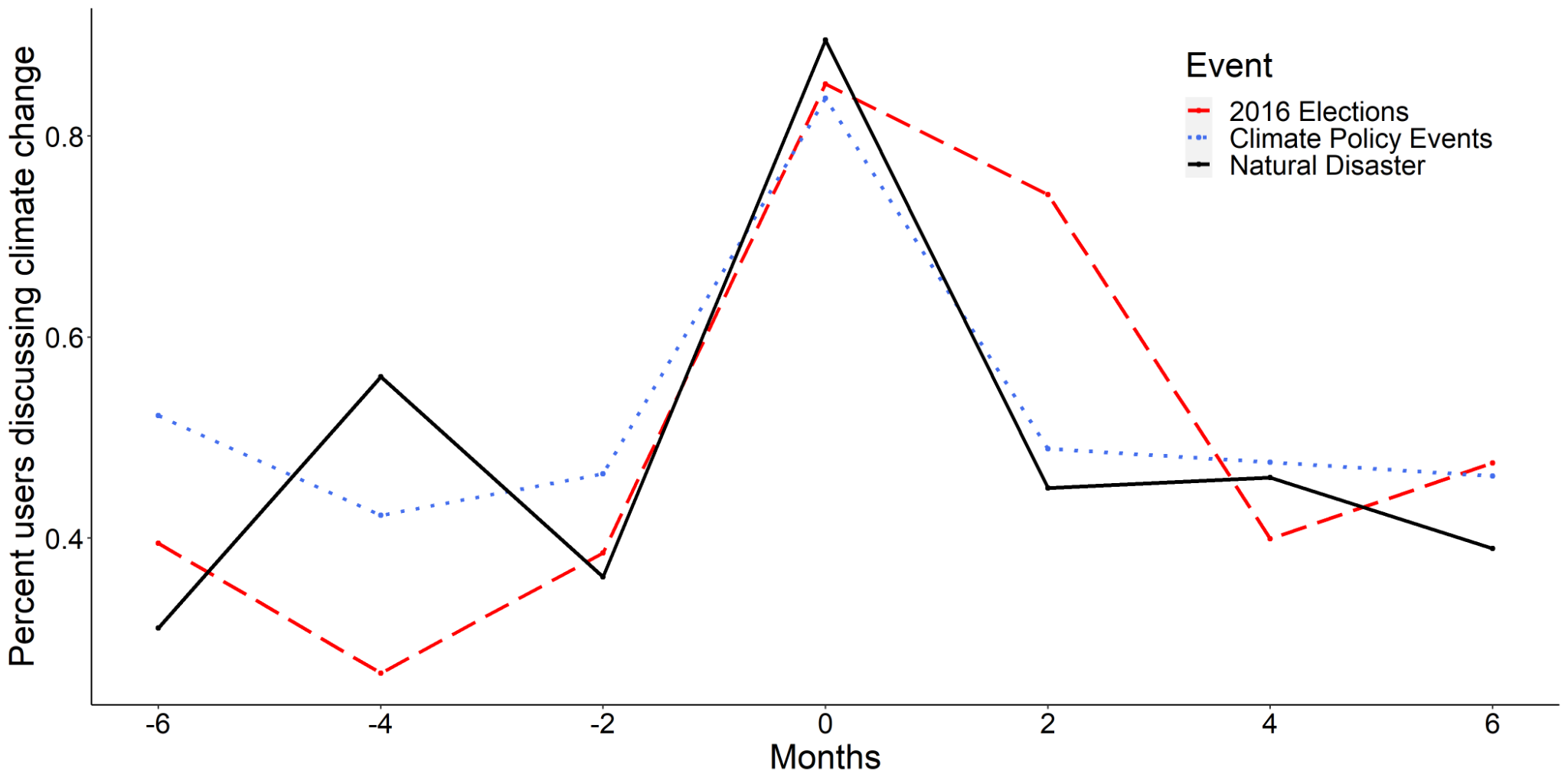
**

**Figure S1.** Bi-monthly rate of distinct users discussing climate change during (1) city-relevant natural disasters (black solid line), (2) the 2016 US Presidential elections (red dashed line) and (3) climate-related policy events (blue dotted line). Note: Month 0 refers to the month of the event occurring.

**References**

[Ryan Broderick. (2018, September 24). *Reddit’s Largest Pro-Trump Subreddit Appears To Have Been Targeted By Russian Propaganda For Years*. BuzzFeed News. https://www.buzzfeednews.com/article/ryanhatesthis/reddits-largest-pro-trump-subreddit-appears-to-have-been](https://www.zotero.org/google-docs/?wrxndJ)
